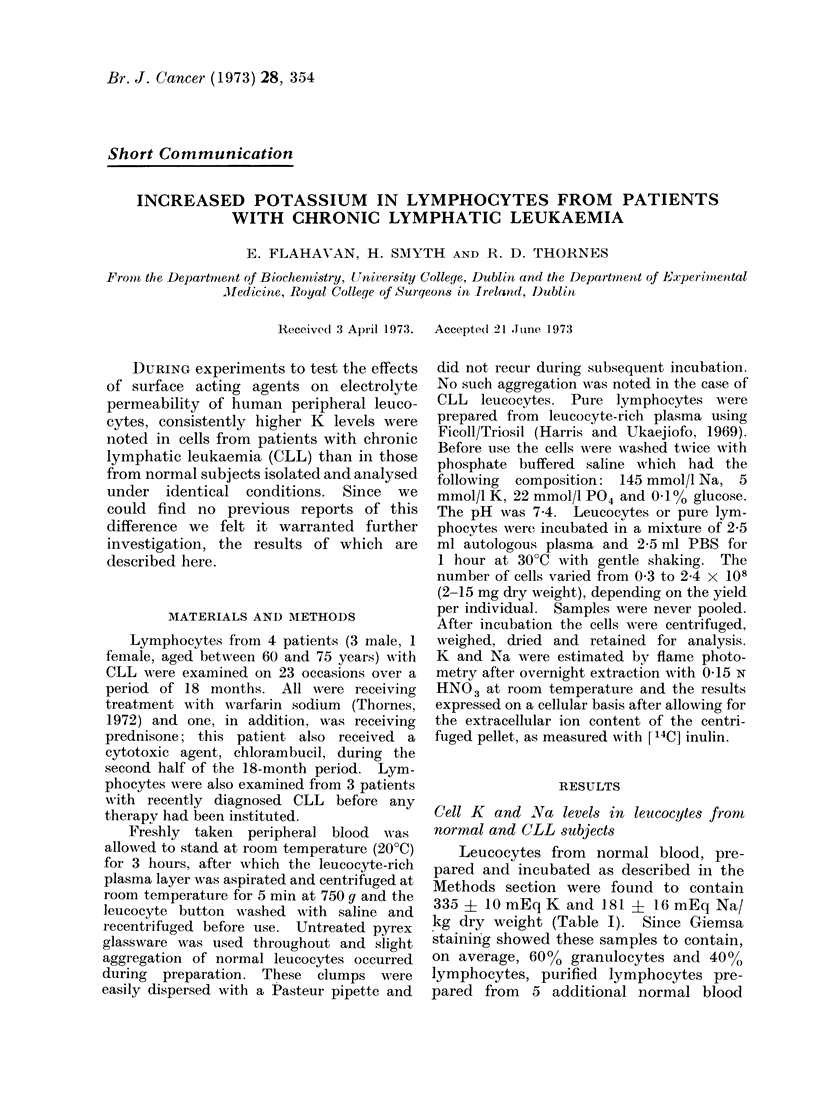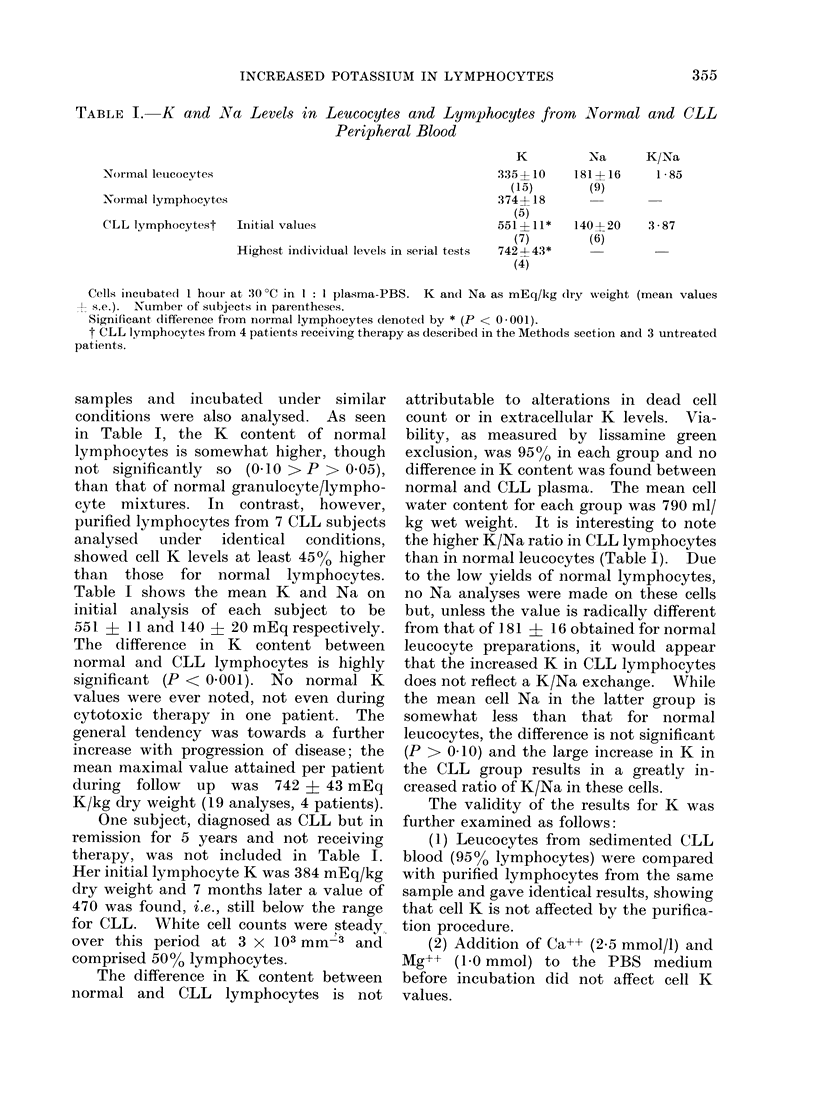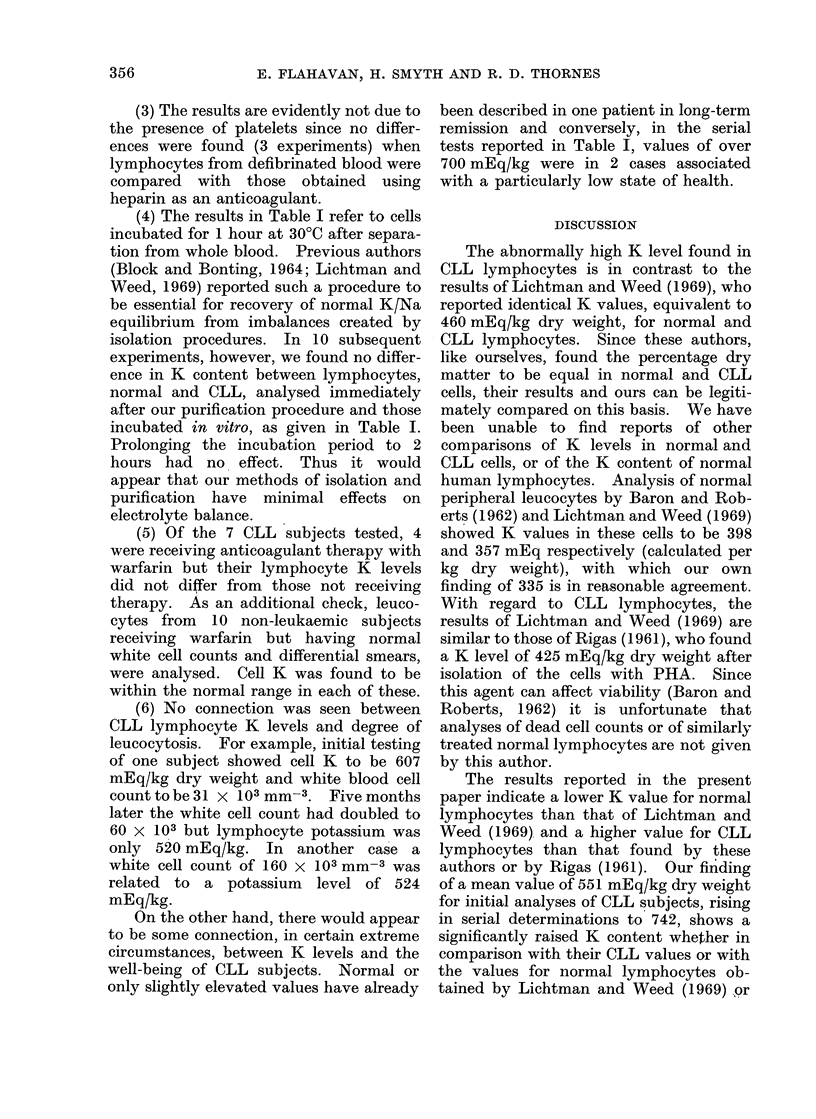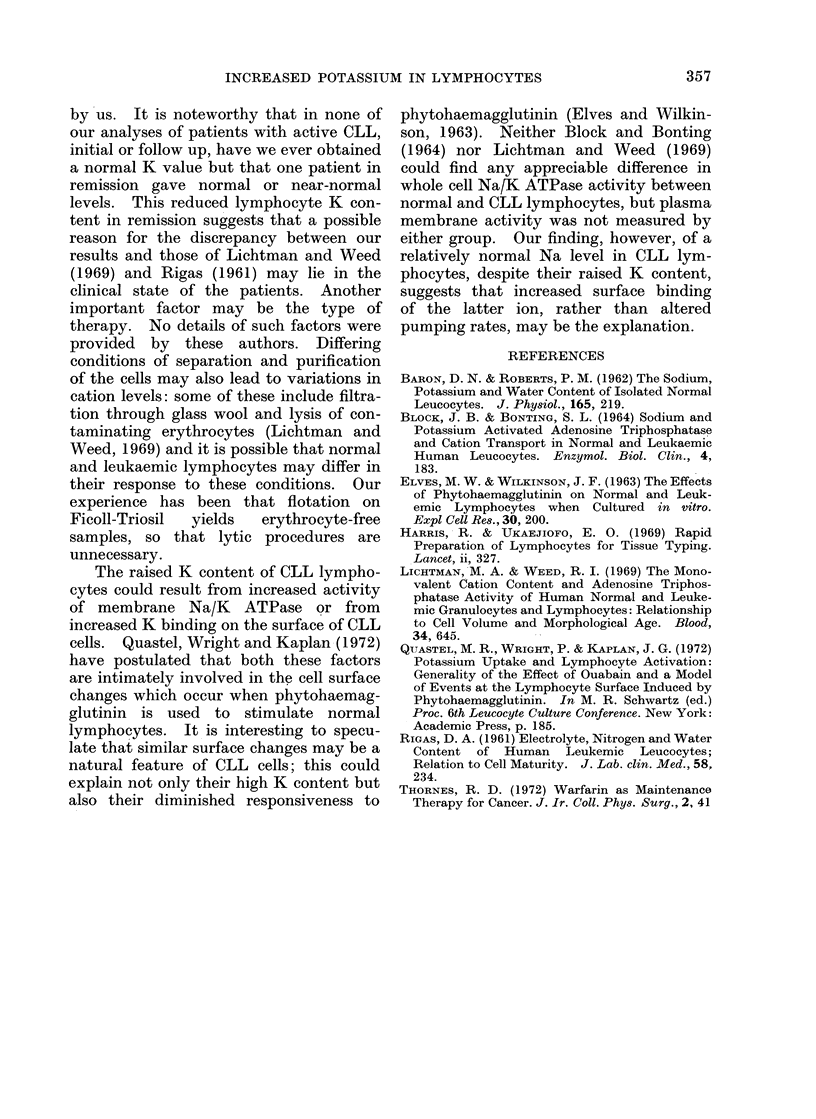# Increased potassium in lymphocytes from patients with chronic lymphatic leukaemia.

**DOI:** 10.1038/bjc.1973.159

**Published:** 1973-10

**Authors:** E. Flahavan, H. Smyth, R. D. Thornes


*Br. J. Cancer* (1973) **28**, 354

***Short Communication***

### INCREASED POTASSIUM IN LYMPHOCYTES FROM PATIENTS WITH CHRONIC LYMPHATIC LEUKAEMIA

E. FLAHAVAN, H. SMYTH AND R. D. THORNES

*From the Department of Biochemistry, University College, Dublin and the Department of Experimental Medicine, Royal College of Surgeons in Ireland, Dublin*

Received 3 April 1973. Accepted 21 June 1973

DURING experiments to test the effects of surface acting agents on electrolyte permeability of human peripheral leucocytes, consistently higher K levels were noted in cells from patients with chronic lymphatic leukaemia (CLL) than in those from normal subjects isolated and analysed under identical conditions. Since we could find no previous reports of this difference we felt it warranted further investigation, the results of which are described here.

### MATERIALS AND METHODS

Lymphocytes from 4 patients (3 male, 1 female, aged between 60 and 75 years) with CLL were examined on 23 occasions over a period of 18 months. All were receiving treatment with warfarin sodium (Thornes, 1972) and one, in addition, was receiving prednisone; this patient also received a cytotoxic agent, chlorambucil, during the second half of the 18-month period. Lymphocytes were also examined from 3 patients with recently diagnosed CLL before any therapy had been instituted.

Freshly taken peripheral blood was allowed to stand at room temperature (20°C) for 3 hours, after which the leucocyte-rich plasma layer was aspirated and centrifuged at room temperature for 5 min at 750 *g* and the leucocyte button washed with saline and recentrifuged before use. Untreated pyrex glassware was used throughout and slight aggregation of normal leucocytes occurred during preparation. These clumps were easily dispersed with a Pasteur pipette and did not recur during subsequent incubation. No such aggregation was noted in the case of CLL leucocytes. Pure lymphocytes were prepared from leucocyte-rich plasma using Ficoll/Triosil (Harris and Ukaejiofo, 1969). Before use the cells were washed twice with phosphate buffered saline which had the following composition: 145 mmol/l Na, 5 mmol/l K, 22 mmol/l $PO_4$ and 0·1% glucose. The pH was 7·4. Leucocytes or pure lymphocytes were incubated in a mixture of 2·5 ml autologous plasma and 2·5 ml PBS for 1 hour at 30°C with gentle shaking. The number of cells varied from 0·3 to $2{\cdot}4 \times 10^8$ (2–15 mg dry weight), depending on the yield per individual. Samples were never pooled. After incubation the cells were centrifuged, weighed, dried and retained for analysis. K and Na were estimated by flame photometry after overnight extraction with 0·15 N $HNO_3$ at room temperature and the results expressed on a cellular basis after allowing for the extracellular ion content of the centrifuged pellet, as measured with [$^{14}C$] inulin.

### RESULTS

### *Cell K and Na levels in leucocytes from normal and CLL subjects*

Leucocytes from normal blood, prepared and incubated as described in the Methods section were found to contain $335 \pm 10$ mEq K and $181 \pm 16$ mEq Na/kg dry weight (Table I). Since Giemsa staining showed these samples to contain, on average, 60% granulocytes and 40% lymphocytes, purified lymphocytes prepared from 5 additional normal blood

TABLE I.—*K and Na Levels in Leucocytes and Lymphocytes from Normal and CLL Peripheral Blood*

| | | K | Na | K/Na |
|---|---|---|---|---|
| Normal leucocytes | | 335 ± 10 (15) | 181 ± 16 (9) | 1·85 |
| Normal lymphocytes | | 374 ± 18 (5) | — | — |
| CLL lymphocytes† | Initial values | 551 ± 11* (7) | 140 ± 20 (6) | 3·87 |
| | Highest individual levels in serial tests | 742 ± 43* (4) | — | — |

Cells incubated 1 hour at 30 °C in 1 : 1 plasma-PBS. K and Na as mEq/kg dry weight (mean values ± s.e.). Number of subjects in parentheses.
Significant difference from normal lymphocytes denoted by * ($P < 0·001$).
† CLL lymphocytes from 4 patients receiving therapy as described in the Methods section and 3 untreated patients.

samples and incubated under similar conditions were also analysed. As seen in Table I, the K content of normal lymphocytes is somewhat higher, though not significantly so ($0·10 > P > 0·05$), than that of normal granulocyte/lymphocyte mixtures. In contrast, however, purified lymphocytes from 7 CLL subjects analysed under identical conditions, showed cell K levels at least 45% higher than those for normal lymphocytes. Table I shows the mean K and Na on initial analysis of each subject to be 551 ± 11 and 140 ± 20 mEq respectively. The difference in K content between normal and CLL lymphocytes is highly significant ($P < 0·001$). No normal K values were ever noted, not even during cytotoxic therapy in one patient. The general tendency was towards a further increase with progression of disease; the mean maximal value attained per patient during follow up was 742 ± 43 mEq K/kg dry weight (19 analyses, 4 patients).

One subject, diagnosed as CLL but in remission for 5 years and not receiving therapy, was not included in Table I. Her initial lymphocyte K was 384 mEq/kg dry weight and 7 months later a value of 470 was found, *i.e.*, still below the range for CLL. White cell counts were steady over this period at $3 \times 10^3$ mm$^{-3}$ and comprised 50% lymphocytes.

The difference in K content between normal and CLL lymphocytes is not attributable to alterations in dead cell count or in extracellular K levels. Viability, as measured by lissamine green exclusion, was 95% in each group and no difference in K content was found between normal and CLL plasma. The mean cell water content for each group was 790 ml/kg wet weight. It is interesting to note the higher K/Na ratio in CLL lymphocytes than in normal leucocytes (Table I). Due to the low yields of normal lymphocytes, no Na analyses were made on these cells but, unless the value is radically different from that of 181 ± 16 obtained for normal leucocyte preparations, it would appear that the increased K in CLL lymphocytes does not reflect a K/Na exchange. While the mean cell Na in the latter group is somewhat less than that for normal leucocytes, the difference is not significant ($P > 0·10$) and the large increase in K in the CLL group results in a greatly increased ratio of K/Na in these cells.

The validity of the results for K was further examined as follows:

(1) Leucocytes from sedimented CLL blood (95% lymphocytes) were compared with purified lymphocytes from the same sample and gave identical results, showing that cell K is not affected by the purification procedure.

(2) Addition of $Ca^{++}$ (2·5 mmol/l) and $Mg^{++}$ (1·0 mmol) to the PBS medium before incubation did not affect cell K values.

(3) The results are evidently not due to the presence of platelets since no differences were found (3 experiments) when lymphocytes from defibrinated blood were compared with those obtained using heparin as an anticoagulant.

(4) The results in Table I refer to cells incubated for 1 hour at 30°C after separation from whole blood. Previous authors (Block and Bonting, 1964; Lichtman and Weed, 1969) reported such a procedure to be essential for recovery of normal K/Na equilibrium from imbalances created by isolation procedures. In 10 subsequent experiments, however, we found no difference in K content between lymphocytes, normal and CLL, analysed immediately after our purification procedure and those incubated *in vitro*, as given in Table I. Prolonging the incubation period to 2 hours had no effect. Thus it would appear that our methods of isolation and purification have minimal effects on electrolyte balance.

(5) Of the 7 CLL subjects tested, 4 were receiving anticoagulant therapy with warfarin but their lymphocyte K levels did not differ from those not receiving therapy. As an additional check, leucocytes from 10 non-leukaemic subjects receiving warfarin but having normal white cell counts and differential smears, were analysed. Cell K was found to be within the normal range in each of these.

(6) No connection was seen between CLL lymphocyte K levels and degree of leucocytosis. For example, initial testing of one subject showed cell K to be 607 mEq/kg dry weight and white blood cell count to be $31 \times 10^3$ mm$^{-3}$. Five months later the white cell count had doubled to $60 \times 10^3$ but lymphocyte potassium was only 520 mEq/kg. In another case a white cell count of $160 \times 10^3$ mm$^{-3}$ was related to a potassium level of 524 mEq/kg.

On the other hand, there would appear to be some connection, in certain extreme circumstances, between K levels and the well-being of CLL subjects. Normal or only slightly elevated values have already been described in one patient in long-term remission and conversely, in the serial tests reported in Table I, values of over 700 mEq/kg were in 2 cases associated with a particularly low state of health.

### DISCUSSION

The abnormally high K level found in CLL lymphocytes is in contrast to the results of Lichtman and Weed (1969), who reported identical K values, equivalent to 460 mEq/kg dry weight, for normal and CLL lymphocytes. Since these authors, like ourselves, found the percentage dry matter to be equal in normal and CLL cells, their results and ours can be legitimately compared on this basis. We have been unable to find reports of other comparisons of K levels in normal and CLL cells, or of the K content of normal human lymphocytes. Analysis of normal peripheral leucocytes by Baron and Roberts (1962) and Lichtman and Weed (1969) showed K values in these cells to be 398 and 357 mEq respectively (calculated per kg dry weight), with which our own finding of 335 is in reasonable agreement. With regard to CLL lymphocytes, the results of Lichtman and Weed (1969) are similar to those of Rigas (1961), who found a K level of 425 mEq/kg dry weight after isolation of the cells with PHA. Since this agent can affect viability (Baron and Roberts, 1962) it is unfortunate that analyses of dead cell counts or of similarly treated normal lymphocytes are not given by this author.

The results reported in the present paper indicate a lower K value for normal lymphocytes than that of Lichtman and Weed (1969) and a higher value for CLL lymphocytes than that found by these authors or by Rigas (1961). Our finding of a mean value of 551 mEq/kg dry weight for initial analyses of CLL subjects, rising in serial determinations to 742, shows a significantly raised K content whether in comparison with their CLL values or with the values for normal lymphocytes obtained by Lichtman and Weed (1969) or by us. It is noteworthy that in none of our analyses of patients with active CLL, initial or follow up, have we ever obtained a normal K value but that one patient in remission gave normal or near-normal levels. This reduced lymphocyte K content in remission suggests that a possible reason for the discrepancy between our results and those of Lichtman and Weed (1969) and Rigas (1961) may lie in the clinical state of the patients. Another important factor may be the type of therapy. No details of such factors were provided by these authors. Differing conditions of separation and purification of the cells may also lead to variations in cation levels: some of these include filtration through glass wool and lysis of contaminating erythrocytes (Lichtman and Weed, 1969) and it is possible that normal and leukaemic lymphocytes may differ in their response to these conditions. Our experience has been that flotation on Ficoll-Triosil yields erythrocyte-free samples, so that lytic procedures are unnecessary.

The raised K content of CLL lymphocytes could result from increased activity of membrane Na/K ATPase or from increased K binding on the surface of CLL cells. Quastel, Wright and Kaplan (1972) have postulated that both these factors are intimately involved in the cell surface changes which occur when phytohaemagglutinin is used to stimulate normal lymphocytes. It is interesting to speculate that similar surface changes may be a natural feature of CLL cells; this could explain not only their high K content but also their diminished responsiveness to phytohaemagglutinin (Elves and Wilkinson, 1963). Neither Block and Bonting (1964) nor Lichtman and Weed (1969) could find any appreciable difference in whole cell Na/K ATPase activity between normal and CLL lymphocytes, but plasma membrane activity was not measured by either group. Our finding, however, of a relatively normal Na level in CLL lymphocytes, despite their raised K content, suggests that increased surface binding of the latter ion, rather than altered pumping rates, may be the explanation.

### REFERENCES

Baron, D. N. & Roberts, P. M. (1962) The Sodium, Potassium and Water Content of Isolated Normal Leucocytes. *J. Physiol.*, **165**, 219.

Block, J. B. & Bonting, S. L. (1964) Sodium and Potassium Activated Adenosine Triphosphatase and Cation Transport in Normal and Leukaemic Human Leucocytes. *Enzymol. Biol. Clin.*, **4**, 183.

Elves, M. W. & Wilkinson, J. F. (1963) The Effects of Phytohaemagglutinin on Normal and Leukemic Lymphocytes when Cultured *in vitro*. *Expl Cell Res.*, **30**, 200.

Harris, R. & Ukaejiofo, E. O. (1969) Rapid Preparation of Lymphocytes for Tissue Typing. *Lancet*, ii, 327.

Lichtman, M. A. & Weed, R. I. (1969) The Monovalent Cation Content and Adenosine Triphosphatase Activity of Human Normal and Leukemic Granulocytes and Lymphocytes: Relationship to Cell Volume and Morphological Age. *Blood*, **34**, 645.

Quastel, M. R., Wright, P. & Kaplan, J. G. (1972) Potassium Uptake and Lymphocyte Activation: Generality of the Effect of Ouabain and a Model of Events at the Lymphocyte Surface Induced by Phytohaemagglutinin. *In* M. R. Schwartz (ed.) *Proc. 6th Leucocyte Culture Conference*. New York: Academic Press, p. 185.

Rigas, D. A. (1961) Electrolyte, Nitrogen and Water Content of Human Leukemic Leucocytes; Relation to Cell Maturity. *J. Lab. clin. Med.*, **58**, 234.

Thornes, R. D. (1972) Warfarin as Maintenance Therapy for Cancer. *J. Ir. Coll. Phys. Surg.*, **2**, 41